# How and Why Patients Adhere to a Prescribed Cardiac Rehabilitation Program: A Longitudinal Phenomenological Study of Patients with Acute Coronary Syndrome

**DOI:** 10.3390/ijerph19031482

**Published:** 2022-01-28

**Authors:** Navin Kaushal, Donya Nemati, Raphaëlle Gauthier-Bisaillon, Marie Payer, Béatrice Bérubé, Martin Juneau, Louis Bherer

**Affiliations:** 1 Department of Health Sciences, School of Health & Human Sciences, Indiana University, Indianapolis, IN 47405, USA; dnemati@iu.edu; 2 Montreal Heart Institute, Montreal, QC H1T 1C8, Canada; rgauthierbisaillon@gmail.com (R.G.-B.); payer.marie@courrier.uqam.ca (M.P.); berube.beatrice.2@courrier.uqam.ca (B.B.); martin.juneau@icm-mhi.org (M.J.); louis.bherer@umontreal.ca (L.B.); 3 Department of Psychology, Universite du Quebec a Montreal, Montreal, QC H3C 5J9, Canada; 4 Department of Medicine, Universite de Montreal, Montreal, QC H3T 1J4, Canada; 5 Centre de Recherche de l’Institut Universitaire de Geriatrie de Montreal, Montreal, QC H3W 1W5, Canada

**Keywords:** qualitative, phenomenology, cardiac rehabilitation, exercise, physical activity, self-determination, autonomous, motivation, theory

## Abstract

**Background.** Adherence to cardiac rehabilitation remains a challenge despite established evidence that engaging in regular exercise is a strong preventive measure to experiencing a second cardiac event. A recent study found a six-month cardiac rehabilitation program to be effective for facilitating regular exercise behavior among patients diagnosed with acute coronary syndrome. The purpose of this study was to conduct a phenomenological investigation using Colaizzi’s descriptive technique to understand mechanisms responsible for behavior change. **Methods.** Data were collected and analyzed among patients with acute coronary syndrome at a cardiac rehabilitation using semi-structured interviews that were conducted over the phone across three months. **Conclusion.** Thematic analysis of 15 semi-structured interviews resulted in 124 statements that were analyzed. The data yielded seven themes that included “motivation to follow prescribed exercise program”, “volitional decision”, “capability of performing exercise”, “connectedness to peers”, “planning”, “habit formation”, and “adopting healthy behaviors beyond exercise”. The emerged themes align with construct definitions of the self-determination theory, which include the three psychological needs (autonomy, competence, and relatedness), in addition to autonomous motivation, which represents internally driven reasons to participate in exercise. Planning and habit formation themes support contemporary research that identifies these constructs responsible for behavioral maintenance. While these themes help explain exercise participation, the final theme, adopting healthy behaviors beyond exercise, reflects the impact of the program on having a change towards a healthier lifestyle. The findings highlight the complexity of exercise behavior, and that long-term participation is likely explained by amalgamating the self-determination theory.

## 1. Introduction

Cardiovascular disease accounts for approximately 30% of deaths annually, making it the leading cause of mortality [1]. The term cardiovascular disease refers to a group of disorders related to the heart and blood vessels. A prevalent form of cardiovascular disease is acute coronary syndrome (ACS), which is a sudden decrease or reduction in blood flow to the heart, which can consequently lead to myocardial infarction. While survival rates of initial cardiac events have improved over the years [2], the event is highly predictive of a second myocardial infarction, which has a much lower survival rate [2]. This is alarming since one in five people who experience a myocardial infarction will experience another event within five years [3]. These findings point to the importance of adopting preventive measures among individuals at risk of experiencing a second cardiac event.

Exercising regularly is a preventive behavior that has been shown to offset a second myocardial infarction, specifically among those caused by ACS [1]. Patients with coronary disease who regularly attended a cardiac rehabilitation program that focused on prescribing exercise have been shown to reduce all-cause mortality by approximately 47% [4,5]. While the importance of facilitating exercise participation among this demographic is acknowledged in the literature [2,6,7,8], behavioral maintenance remains a challenge as dropout rates in cardiac rehabilitation programs average 60% among 37 countries [9].

A recent randomized controlled pilot trial tested the effectiveness of facilitating exercise participation among patients with ACS at a cardiac rehabilitation center over six months. The trial comprised a quantitative and qualitative investigation. The quantitative components were recently published, which also includes details on the trial design [10]. In summary, participants in the experimental arm attended monthly presentations (workshops) from months 1–3 and received phone call follow-ups from months 4–6. Participants were primarily guided on how to establish a preparatory exercise habit, which is defined as a phase that encompasses activities that readies oneself to exercise (e.g., preparing a water bottle and gym clothes) [11]. Specifically, participants were instructed to exercise at least four days per week, as that has been shown to be the minimum behavioral requirement to establish an exercise habit [12]. Additionally, participants were informed on the importance of establishing temporal consistency and cues. For temporal consistency, participants were advised to schedule the same pattern of behavioral activities to establish predictability (e.g., perform morning routine, then work out) [13]. Participants were advised to implement cues by (i) making them salient and (ii) turning the cue on (to prompt behavior) and off (once the behavior has been performed) [13]. For instance, a cue can be activated by placing gym clothes on the bed in the morning. The clothes will remain on their bed until it is time for their workout, where the participant would wear the clothes then put them away in their closet to turn off the cue. 

Additional details regarding the presentation have been previously reported [10]. Participants were then monitored and contacted via a phone call during the maintenance phase (months 4–6) to assess if there were any safety concerns and to conduct semi-structured interviews. Quantitative analyses of this study demonstrated that the intervention was effective in increasing weekly exercise engagement time; however, the mechanisms which address *what* caused behavior change, and *how* behavior change remained to be addressed via a qualitative approach. This is vital as behavior change is complex and there are several determinants that can contribute to understanding behavior change or increasing exercise participation [14]. The purpose of this study was to identify mechanisms that were determinants of facilitating exercise participation in the program.

## 2. Materials and Methods

### 2.1. Study Design

The objective of the study was to develop a qualitative phenomenological methodology to understand the lived experience of patients with ACS in the exercise intervention arm. The phenomenological approach seeks to uncover “what makes something what it is” [15], by scrutinizing the experiences that patients lived through retrospectively [16]. Phenomenological investigation identifies the meaning, structure, and essence of the consciously lived experience of a phenomenon (e.g., developing an exercise habit) among a specific demographic, or individuals with the same characteristics with a shared experience [17]. This is relevant because participants with the same diagnosis (ACS) were having the same shared experience of participating in exercise in the same environment. Capturing how participants experience, interpret, describe, remember, and judge the phenomenon of interest necessitates investigation of research questions by qualitative investigation [18,19]. The goal of phenomenology is to unveil the essential structure of experience while avoiding interpretation, explanations, and adding to or subtracting from the lived experience [20,21]. Therefore, this study’s main objective was to describe the lived experience of patients with ACS of adhering to a cardiac rehabilitation program. This methodology is a rigorous and well-established approach found in cardiac rehabilitation research [22,23], including a recent systematic review [24].

Participants (n = 13) were older adults (50–85 years old), diagnosed with acute coronary syndrome, and were prescribed with cardiac rehabilitation at the Montreal Heart Institute, with five participants who were randomized to the intervention arm. Potential eligible participants received a study advertisement from a nurse at the cardiac rehabilitation and were enrolled on a rolling basis. Only one participant from the experimental arm dropped out after providing consent and prior to attending any study sessions. The study was delivered, and data were collected at the cardiac rehabilitation center. All participants in the intervention arm participated in monthly interviews for three consecutive months, totaling 15 interviews. Participants were 69.6 (SD = 5.60) years of age, composed of 80% females, and were engaging in 56.80 (SD = 84.67) min of moderate-to-vigorous physical activity/week. The overall status of health, which included cardiac-related symptoms, were slightly above average. Complete demographic characteristics have been previously reported [10]. The sample size was deemed to be valid based on several recommendations on conducting a phenomenological investigation, in addition to similarities of related studies. First, a general recommendation is for qualitative studies to have 3–15 participants [25]. Second, given the recognized, large dropout rate in cardiac rehabilitation, studying participants who demonstrated an exercise habit for six months is rare, but vital, as this is the goal of cardiac rehabilitation, and is the phenomenon of interest [26,27]. Since these participants were successful in their experience, they accumulated substantial knowledge of their experience for a richer discussion, which lowers the needed sample size [28]. Finally, the study was a pilot design that followed sample selection with similar longitudinal studies [22,29]. However, the present study was distinct from previous related work as the data were from a behavioral (exercise) intervention that resulted in 15 interviews that yielded a saturation of themes and subthemes. To identify the appropriate number of participants for our purpose, we followed the suggestion by Guest et al. (2001), who stated that “the magic number of six interviews is consistent with Morse’s (1994) (albeit unsubstantiated) recommendation for phenomenological studies” [30]. Other researchers recommended ten [31] and thirteen [32] interviews to be a safe threshold. The present study analyzed a total of 15 interviews that comprised 124 transcribable statements, thus meeting sample size requirements across several proposed primers. 

### 2.2. Data Collection

Qualitative data from semi-structured interviews were obtained based on recommended guidelines [25,33]. Participants were provided with a worksheet during their first meeting to guide them in establishing an exercise habit that included a preparation process, scheduling a time, consistency, and cues. Asking participants to reflect on the worksheet provides direction and flexibility for conducting semi-structured interviews. Participants completed a total of three interviews, and interview questions were generated by the PI. The interviews ranged from 15–25 min in length and were audio-recorded and included field notes. The transcripts were returned to participants to confirm if the interpretations were accurate. 

### 2.3. Data Analysis

The rationale for the study led to the application of Colaizzi’s descriptive phenomenological technique as a theoretical framework and analytical method [34]. Identifiable information from participants were removed before transcription. In the first step of In Colaizzi’s descriptive data analysis technique, significant statements are extracted that pertain to the phenomenon under inquiry which was developing an exercise habit in a cardiac rehabilitation setting [35]. After reading and listening to the recorded audios multiple times, significant statements were identified for each participant. Re-statements were created, and the meaning was formulated for all significant statements. Coding from the two reviewers (R.G. and M.P.) was used to ensure agreement in labeling, and any disagreements were discussed until consensus and consistency was reached. Thoughts, ideas, and notes were recorded related to each statement, which led to theme formulation. Validation of identified themes was approved by all authors involved in qualitative analysis. The data were imported into qualitative data analysis software NVivo 12 [36] to code statements and extract themes, theme clusters, and theme categories. 

### 2.4. Trustworthiness and Rigor

To address the trustworthiness of the data, components such as credibility, confirmability, transferability, and dependability were evaluated by the following steps. To guarantee credibility, member checking for data analysis and peer debriefing were assured. For dependability, constant journaling, note-taking, recording thoughts, changes, adjustments, and peer debriefing were performed. Confirmability was conducted by an audit trail, and it was reviewed by the research team. Transferability was addressed by providing the context of data collection, location, participants’ characteristics, and data analysis procedures [37,38]. We also followed the recommended guidelines for conducting qualitative investigation in sport and exercise psychology by Smith & McGannon (2018) to maximize the validity of the findings, which were consistent with guidelines proposed [37,38]. Table 1 outlines how we applied the “top 10 rigor/trustworthiness techniques” to the present study [39]. The interviews were facilitated by M.P. (BSc), who is a female, doctoral student trained by the PI (N.K.) on how to conduct the interviews. M.P did not establish a relationship with any participant prior to the study. The interviewer only provided a professional introduction to each participant, which included her research area in cardiac rehabilitation. The study follows the consolidated criteria for reporting qualitative research (COREQ) guidelines [40] (Table S1).

## 3. Results

Seven themes emerged from the data, which can be found in Table 2. The first theme to emerge was “motivation to follow prescribed exercise program”, where participants discussed the sources of their motivational drive that facilitated consistent engagement. These sources were categorized into two subthemes which were affective motivation and personal health outcomes. The next theme was “volitional decision”, which comprised statements where participants exercised autonomy to reach their goals, which included selecting their preferred forms of exercise, and performing self-initiated goal-oriented actions that were not instructed in the study program. The third and fourth theme to emerge was “capability of performing exercise” and “connectedness to peers”, which reflected proficiency and building relationships with their peers in the rehabilitation program, respectively. The fourth theme was “planning” and the fifth theme was “habit formation”. Finally, the last theme to emerge was “adopting healthy behaviors beyond exercise”. These themes and subthemes are elaborated upon below. 

### 3.1. Motivation to Follow the Prescribed Exercise Program

Motivation was the most prominent theme that emerged from the data, which was defined as the reasons for performing exercise. Sources of motivation were *affective motivation* and *personal health outcomes.* We define affective motivation as an intrinsic, positive feeling about performing exercise. Some examples of affective motivation included the following statements:

*“I feel self-motivated. Sometimes my schedule is pretty busy. But when I return to the (swimming) pool, I remember that I really enjoy it.”*

(interview 3) [P01].

Participant two also reported being motivated by *personal health outcomes* and the remaining three participants provided examples that were themed as identified motivation:

*“Results of my blood tests motivated me to continue attending the (rehabilitation) center.”*

(interview 1) [P02].

*“I noticed I lost a lot of weight and I feel motivated to work on strength and endurance training.”*

(interview 3) [P03].

### 3.2. Volitional Decision

All participants were found to report a form of action that was coded as volitional decision. These decisions included modifying their schedule and exercise preference; specifically, subthemes of volitional that emerged included *scheduling, exercise preferences, and self-initiated actions beyond the prescribed program* that facilitated the development of their exercise behavior. For instance, three participants expressed that they changed their *schedule* accordingly:

*“My initial goal was to attend (the gym) three times/week, but I changed it to twice per week. It has been more realistic for my schedule.”*

(interview 2) [P01].

*“My schedule is not consistent, so I change my exercise timings to fit.”*

(interview 3) [P04].

Participants discussed that they enjoyed selecting their workouts:

*“I am not in a group, but when I see an interesting one I join.”*

(interview 2) [P05].

*“In the long run, I feel motivated because I only participate in exercise (that) I like, so I stick with those that I find enjoyable…it keeps me motivated.”*

(interview 1) [P01].

Finally, two participants commented on implementing self-initiated actions beyond the exercise program to help facilitate their workouts.

*“I bought a rubber stretch mat that I like to take with me.”*

(interview 1) [P02].

*“I want to make some memory aid after the trainings to be able to reproduce the exercises at home.”*

(interview 2) [P03].

### 3.3. Capability of Performing Exercise

“Capability” of performing exercise emerged from the data, which included competency in learning how to efficiently plan and perform their workout. For instance, participant 04 found that the workshop helped him to improve his confidence while working out, “*I found the workshop to help me feel more confident about exercising*” (interview 1) [P04]. Another example included evidence of moving towards exercise independence. For instance, participant 03 gained the confidence to move towards strength/exercise training after observing health outcomes from cardiovascular exercise, “*I noticed I lost a lot of weight and I feel motivated to work on strength and endurance training*” (interview 3) [P03]. The data of participant 05 showed a transition from prescribed, supervised exercise session towards being physically active on his own by joining a volleyball team, “*I always wanted to try volleyball … it’s fun and I plan to continue playing*” (interview 3) [P05].

### 3.4. Connectedness to Peers

The theme of “connected to peers” emerged from statements related to the experience of pleasure when exercising with other members at the rehabilitation facility. One of them created friends during the study, “*It’s easier with friends when you need to continue a (regular) training pace*” (interview 2) [P01], while the other indicated his interest in developing an exercise group, “*I would like to develop a small network to meet at the gym*” (interview 2) [P04].

### 3.5. Planning

The “Planning” theme, which emerged from the data that supported the importance of regular usage of a planner (worksheet), was found across all participants. All participants emphasized the role of planner as a cue to action:

*“I use my planner to help maintain my training schedule.”*

(meeting 1) [P01].

*“I found the planner to be a helpful tip presented at the meeting.”*

(interview 2) [P04].

*“Being consistent was important to me, and my planner helped.”*

(interview 2) [P05].

### 3.6. Habit Formation

The theme of habit merged from statements that referred to successful behavior if they included less reliance on conscious regulation and categorized in three subthemes that included “progress towards automaticity”, “consistency of preparation”, and “using cues to prompt behavior”.

(i) Progress towards automaticity

*“I don’t need to make any changes. My workout habit has been established after three months.”*

(interview 1) [P05].

For instance, [P05] mentioned that he no longer checks his planner:

*“it’s in my head….it has become a habit.”*

(interview 3) [P05].

(ii) Consistency of preparation

Consistency of preparation was defined as being prepared to work out at the planned time. Examples are provided below:

*“Having my sport bag ready was a helpful note (from the presentation) that was a solution to my obstacles.”*

(interview 3) [P01].

P05 explained that he maintains regularity in his training so it is easier for him to remember, “I maintain consistency in my training. I go for my workouts at the same time” (interview 2) [P05].

(iii) Using cues to prompt behavior

“Cues” were defined as something associated with exercise that prompts the initiation of behavior. Three participants reported the incorporation of cues to prompt their exercise sessions. One participant (P04) resorted to using his cellphone as a reminder, while three participants used their exercise gear:

*“I keep my sport bag ready for the gym.”*

(interview 3) [P01].

*“The worksheet was useful: my bag, and all my training gear is ready when the activity is scheduled.”*

(interview 1) [P03].

### 3.7. Adopting Healthy Behaviors beyond Exercise

Three participants found improvements in other health-related behaviors as a direct result of transferring skills learned from the program meetings that potentially improved overall wellbeing. The following are some examples that support this theme:

*“I used the worksheet to also make sure I eat my lunch on time. Keeps things easy”*

(interview 1) [P01].

*“I am motivated to improve my health. I check my cholesterol on the morning.”*

(interview 2) [P03].

*“I have a new challenge which is to decrease my alcohol intake to have better performance at the gym. I also go out more often, not just to the gym.”*

(interview 3) [P04].

## 4. Discussion

The purpose of this study was to conduct a phenomenological investigation to understand the lived experience among patients with ACS who participated in an exercise program offered at a cardiac rehabilitation center. The investigation was performed by conducting semi-structured individuals monthly, from months 4–6. Colaizzi’s descriptive technique revealed seven core themes that included, motivation to follow a prescribed exercise program, volitional decision, capability of performing exercise, connectedness to peers, planning, habit formation, and adopting healthy behaviors beyond exercise. We found these themes to align with constructs that support a prominent behavior change theory, the self-determination theory [42], in addition to recognizing behavioral maintenance processes.

### 4.1. Self-Determination Theory

The self-determination theory proposes that we have three basic needs, which are determinants of behavioral enactment that include autonomy, competence, and relatedness [43]. Autonomy reflects our ability to make choices when performing a behavior. Competence is defined as the ability, skill, and knowledge necessary to perform the behavior. Finally, relatedness represents a need to be affiliated with others to feel a sense of connection. These needs are also predicted by motivation, which exists on a continuum of autonomous/self-determined (intrinsic, integrated, and identified) to controlled/non-self-determined motivation (introjected, external, and amotivation) at the opposite end of the spectrum [43]. The present study found themes to align with the three basic needs and autonomous motivation.

### 4.2. Autonomous Motivation 

The present study identified the presence of *affective motivation* and *personal health outcomes*, which align with definitions of *intrinsic* regulation (inherent enjoyment) and *identified* regulation (value of the goal), which are forms of autonomous motivation proposed in the self-determination theory [43]. The study program introduced participants to a variety of exercise options that ranged from solo to group workouts, thus allowing them to select exercise options they found to be *intrinsically* enjoyable. The workshops also presented how exercise is a strong preventive measure from experiencing a second myocardial infarction in addition to improving their health-related quality of life. This material may have helped participants facilitate their *identified* regulation where they recognized the self-value of exercising regularly. These findings overall revealed that participants were driven by autonomous instead of a controlled form motivation to engage in regular exercise. Between the two forms, autonomous motivation has been found to facilitate behavioral repetition and prevent dropout because it can be self-sustained as it does not depend on external motivators [42,44], which has been supported by empirical findings [45]. These findings are consistent with previous work that found providing education sessions for cardiac rehabilitation patients can contribute to facilitating their motivation to participate in exercise [24].

### 4.3. Psychological Needs

Three themes (*volitional decision, capability of performing exercise,* and *connectedness to peers*), based on the transcriptions, were found to align with the basic needs from the self-determination theory (autonomy, competence, relatedness, and autonomous motivation), (Deci and Ryan, 2002). Volitional decision, or autonomy, was found to be a prominent theme and the thematic analyses revealed insightful ways on how participants appreciated autonomy, which included the option to select their favorite types of exercise [P01] and scheduling to fit their lifestyle [P02]. Competence (capability of performing exercise) was also strongly supported by participants applying the presented tips from the workshop and the ability to perform exercises. For instance, participants commented on their success in adopting strategies presented in the workshop to facilitate an exercise habit [P04]. A participant also reported moving from group to independent training, specifically from participating exclusively in a guided group-based cardiovascular classes (spin class) to solo endurance and strength training [P03]. The presence of the competence theme carries significant importance among cardiac rehabilitation patients as they are generally physically inactive prior to experiencing a cardiac event, and in a rehabilitation phase, it is vital to help them develop exercise competence to facilitate regular participation. Finally, connectedness to peers, or opportunities to facilitate relatedness, also emerged from the workshops, which allowed participants to interact with others who were also in rehabilitation. The development of these constructs supports previous qualitative investigation on the importance of basic needs from the self-determination theory [46]. Additionally, these findings are supported by a recent systematic review that found feeling connected to peers facilitates autonomous motivation [24]. In summary, these themes support the self-determination theory, and these findings are congruent with a review on cardiac rehabilitation that identified some constructs from this theory to be predictive of participation in exercise among this demographic [47].

### 4.4. Behavioral Maintenance Constructs

Two emerged themes, planning and habit formation, were found to be prominent among participants. These constructs are recognized as behavioral change determinants, and in several contemporary theories are recognized as post-intentional and maintenance constructs [48,49,50]. Habit formation, which relies on temporal consistency and cues [12,13,51], was revealed to help participants engage in exercise regularly. Participants showed greater usage of implementing routine consistency, resulting from planning use [*P05*] and using environmental cues, such as a “cell phone reminder” and “exercise bag” [*P04, P03, P01*], thus corroborating the fidelity of the intervention. The techniques were adopted from previous work that found these strategies to be effective for facilitating an exercise habit among general adults [13]. The findings of this study provide encouraging notes to test the emerged themes in a larger scale for patients with acute coronary syndrome, and for patients with other conditions who have been prescribed to cardiac rehabilitation. Participants were also guided by their worksheets on how to develop their plan, which emerged as a theme that was favorable and effective in exercise participation. Planning helps prepare and protects a timeslot for exercise, which has been recognized to be vital, particularly among novice exercisers [52]. Planning is especially important among clinical population groups because identified reasons for dropouts include irregularity (lack of consistency), low automaticity, and self-regulation (planning) [53], which reflect the importance and the presence of these variables in this study. While investigations are limited among individuals with acute coronary syndrome, planning has been found to be an effective behavior change technique for people with heart failure [54].

### 4.5. Adopting Healthy Behaviors beyond Exercise

The final emerged theme was adopting healthy behaviors beyond exercise. This theme was supported by transcripts of participants becoming more conscious of their diet, such as aiming to incorporate more vegetables, having as a goal to decrease alcohol intake, and monitoring their cholesterol levels every morning. Interestingly, these transcripts revealed that some who facilitated regular exercise behavior also began to monitor and control unhealthy behavior, such as decreasing alcohol intake [P04]. Self-endorsed choices for making changes in health behaviors is a phenomenon that has been identified among other rehabilitation populations (such as an increase in exercise activity and decrease in smoking) [55,56], though exact underlying behavioral mechanics that cause this effect are not clear. 

### 4.6. Strengths and Limitations 

Although the study demonstrated efficacy for adopting novel behavioral tactics for individuals with acute coronary syndrome, the findings should be interpreted in a respective scope. While qualitative data provide enriching insights that are not confined to quantitative measurements, this brings a potential limitation of coding, and interpretation of transcripts that could be subjective to the interpreters. Though we recognized this limitation and attempted to prevent this effect by following reporting methodological approaches by “bracketing” or “epoché”, which is an essential step of a descriptive phenomenological approach that focuses on the “pure description of” human experiences rather than interpretation [20,57]. For enhancing the validity of qualitative data analyses, we also used qualitative reporting guidelines [39]. In light of these notes, the study includes notable strengths worth highlighting. The study makes a contribution to the phenomenological literature on understanding the lived experience of cardiac rehabilitation [23,24,26,58]. However, this study is distinguished from previous works, as it was a six-month intervention design that included repeated interviews. Specifically, the intervention facilitated exercise participation during the program, which revealed how the lived experience evolved across time as participants became more comfortable participating in exercise. Finally, the thematic elements are largely supported by a recognized theory, post-intentional processes, and other notable constructs that are relevant for this demographic.

## 5. Conclusions

The phenomenological investigation revealed themes supporting self-determination theory, behavioral maintenance that explained their participation in the rehabilitation program, in addition to behavior transference, which reflected a motivation to adopt and manage other health-related behaviors. The intervention provided a workshop, which is likely absent across other rehabilitation centers, which justified the pilot design. The three-month assistance via the workshop program demonstrated to be a resource for developing determinants for successful behavioral adoption and maintenance. The identified themes provide translational notes on components to include for rehabilitating patients diagnosed with ACS. Specifically, guiding participants on completing a habit formation worksheet that helped them establish temporal consistency and cues along with planning to work out for at least four days per week, helped stabilize and protect their schedule. Additionally, delivering a program based on the tenets of the self-determination theory that includes giving them autonomy regarding their exercise schedule with opportunities to develop competence, and access to a supportive environment (relatedness), demonstrated to be effective mechanisms to help the participants sustain their exercise behavior. Overall, the findings highlight the complexity of exercise behavior, and that long-term participation is likely explained by amalgamating the self-determination theory by including habit and planning tactics. Future research designs that implement these determinants in a full clinical trial is warranted. 

## Figures and Tables

**Table 1:** Application of qualitative techniques for rigor/trustworthiness by McGannon et al., (2019) [39].

**Technique** **Application in the current study**
**1. Interrater reliability**
The statements were independently coded by two different reviewers. Any discrepancies in coding were discussed until mutual agreement was achieved
**2. Member checking**
Member checking was performed by reviewing the transcribed statements with each participant
**3. Bracketing/researcher reflexivity**
Potential for bias interviewing was prevented by having a data coder who was a trained qualitative researcher but from a different discipline. Bias was also prevented by ensuring that the PI did not conduct the interview or code the data
**4. Theoretical coherence**
The aim of the study was to gather data of patients’ experience of exercising regularly. The methodological approach, phenomenology, is congruent with this aim as the approach does not attempt to explore hypotheses or causalities, but only attempts to understand [41]
**5. Outsider/expert check**
An outsider (DN), who was not involved in prior stages of the study, reviewed if the results were theoretically sound
**6. Critical friends**
N.K. served as the critical friend for the data collected by R.G., M.P., and B.B.
**7. Researcher background**
All individuals involved in participation interaction (M.P., B.B., and R.G.) provided an introduction of themselves along with their research backgrounds to the participants
**8. Pilot interviews**
The study is a pilot investigation, and so these interviews will support the design of a larger scale study
**9. Multiple methods/triangulation**
Two forms of data were collected from the participants, which included written data (planning sheets) and semi-structured interviews. Some data overlap of these two methods was expected, which helped in triangulating the findings.
**10. Audit trail/transparency**
All the raw participant data, including the coding, and their interpretation are available

**Table 2:** Themes and subthemes from the data analysis.

***Theme* Subtheme *Data Sample***
***1. Motivation to follow a prescribed exercise program***
*I.* *Affective motivation*
*P01* *“In the long run, I can choose the types of activities that I want to do. This makes working out enjoyable and keeps me motivated. (For example), I don’t like running, so I don’t do it.”* (interview 1) *P02* *“I like to go at my own pace. I don’t force myself when I don’t feel well. I also keep doing other things I enjoy (such as), Spanish lessons and cooking.”* (interview 2)
*I.* *Personal health outcomes*
*P03* *“I remember the benefits (of) exercising and how important it is to me, when I don’t feel like going.”* (interview 1) *P01* *P01 “Noticing how I improve over time encourages me. I can now easily climb up my apartment stairs.”* (interview 1)
***2. Volitional decision***
*I.* *Scheduling*
*P02* *“My motivation is high because I like to go at my own pace. I don’t force myself too much, or sacrifice my other hobbies (Spanish lessons and cooking).”* (interview 2)
*II.* *Exercise Preferences*
*P05* *“I mostly do spin classes and floor exercises”…, “I always wanted to try volleyball….it’s fun and I plan to continue playing.”* (interview 3) *P01* *“I don’t really like running so I don’t force myself to do it.”* (interview 1)
*III.* *Self-initiated actions beyond the prescribed program*
*P02* *“I rented a locker at the center so I can keep my clothes there, so I do not have to bring them from home.”* (interview 2)
***3. Capability of performing exercise***
*P04* *“I found the workshop to help me feel more confident about exercising.”* (interview 1) *P03* *“I noticed I lost a lot of weight and I feel motivated to work on strength and endurance training.”* (interview 3) *P05* *“I like exercising with groups (e.g., spin classes)”* (interview 1), *“I always wanted to try volleyball….it’s fun and I plan to continue playing.”* (interview 3)
***4. Connectedness to Peers***
*P01* *“It’s easier with friends when you need to continue a (regular) training pace.”* (interview 2) *P04* *“I would like to develop a small network to meet at the gym.”* (interview 2)
***5. Planning***
*P02* *“(Initially) I use my planner to be organized, “I still use my planner regularly.”* (interview 3) *P03* *“I always use my planner.”* (interview 1) *P04* *“The worksheet worked as a good planner.”* (interview 1) *P05* *“I discovered a new tip—it’s to always use my planner”* (interview 2), *“I don’t use my planner as much because my schedule is in my head, it’s become a habit.”* (interview 1)
***6. Habit formation***
*I.* *Progress towards automaticity*
*P05* *“My new routine is effective. I find that the first three free months helped a lot in creating my routine.”* (interview 1) *P05* *“I no longer check my planner. It’s in my head….it has become a habit”* (interview 1)
*II.* *Consistency of preparation*
*P05* *“Having a routine was a new tip for me. Going to the center at the same times and days of the week is what helped me not stop training.”* (interview 3) *P01* *“I found the tip (from the presentation) about having your bag ready to be helpful.”* (interview 3)
*III.* *Using cues to prompt behavior*
*P03* *“The worksheet was useful: my bag, and all my training gear is ready when the activity is scheduled.”* (interview 1) *P04* *“Discipline myself to always have all the accessories ready for training.”* (meeting 1) *P04* *“I kept a reminder on my cell phone to help remind me.”* (interview 1)
***7. Adopting healthy behaviors beyond exercise***
*P01* *“I used the worksheet to also make sure I eat my lunch on time. Keeps things easy.”* (interview 1) *P04* *“I have been putting a lot of effort to my diet.”* (interview 1) *P04—“to plan meals…, “I started an eating routine that includes more vegetables and less red meat.”* (interview 2)

## Data Availability

The data presented in this study are available on request from the corresponding author. The data are not publicly available due to privacy or ethical restrictions.

## Supplementary Materials

The following are available online at https://www.mdpi.com/article/10.3390/ijerph19031482/s1, Table S1: Consolidated criteria for reporting qualitative studies (COREQ): 32-item checklist.
