# Supplementary material for: How and Why Patients Adhere to a Prescribed Cardiac Rehabilitation Program: A Longitudinal Phenomenological Study of Patients with Acute Coronary Syndrome
Source: Int J Environ Res Public Health. 2022 Jan 28;19(3):1482. doi: 10.3390/ijerph19031482 (PMC8834758; doi:10.3390/ijerph19031482)
Supplement: Supplementary file 1 [file ijerph-19-01482-s001.zip › ijerph-1568662-S1.pdf]

Table S1. Consolidated criteria for reporting qualitative studies (COREQ): 32-item checklist.

Item	Description and Page Number
**Domain 1:Research team and reflexivity**	
1. Interviewer/facilitator	M.P. (page 4)
2. Credentials	BSc (page 4)
3. Occupation	graduate student (page 4)
4. Gender	female (page 4)
5. Experience and training	trained by PI (N.K.) (page 4)
Relationship with participants	
6. Relationship established	no relationship established with participants prior to the study (page 4)
7. Participant knowledge of theinterviewer	professional introductory knowledge (page 4)
8. Interviewer characteristics	the interviewer is interested in cardiac rehabilitation (page 4)
**Domain 2: study design**	
Theoretical framework	
9. Methodological orientation and Theory	phenomenology (page 2)
Participant Selection	
10. Sampling	rolling basis (page 3)
11. Method of approach	referral from nurses (page 3)
12. Sample size	n = 13 (page 2)
13. Non-participation setting	one participant dropped out before attending the first meeting (page 2)
14. Setting of data collection	cardiac rehabilitation center (page 2)
15. Presence of non-participants	no one else present (page 2)
16. Description of sample	patients with acute coronary syndrome (page 2)
Data Collection	
17. Interview guide	the authors generated the questions (page 3)
18. Repeat interview	yes, there were two repeat interviews totaling three interviews (page 3)
19. Audio/visual recording	the researchers used audio recording (page 3)
20. Field notes	field notes were made during the interviews (page 3)
21. Duration	interviews ranged from 15–25 min (page 3)
	saturation reported (page 3)
22. Data Saturation	transcripts were confirmed (page 3)
23. Transcripts returned	transcripts returned (page 3)
**Domain 3: analysis and findings**	
Data analysis	
24. Number of data coders	there were two data coders R.G and M.P(page 4)
25. Description of the coding tree	description of the coding tree (page 2)
26. Derivation of themes	themes were derived from the data (page 4)
27. Software	NVivo 12 was used (page 4)
28. Participant checking	participants did not provide feedback
Reporting	
29. Quotations presented	quotes and participant numbers were provided (Table 2)
30. Data and findings consistent	consistency has been reported (page 4)
31. Clarity of major themes	major themes identified (page 4)
32. Clarity of minor themes	minor/subthemes identified (page 4)
